# LPS-induced macrophage HMGB1-loaded extracellular vesicles trigger hepatocyte pyroptosis by activating the NLRP3 inflammasome

**DOI:** 10.1038/s41420-021-00729-0

**Published:** 2021-11-06

**Authors:** Guozhen Wang, Siyi Jin, Weichang Huang, Yang Li, Jun Wang, Xuguang Ling, Yun Huang, Ye Hu, Congcong Li, Ying Meng, Xu Li

**Affiliations:** 1 Department of Emergency Medicine, Nanfang Hospital, Southern Medical University, 510515 Guangzhou, China; 2 State Key Laboratory of Organ Failure Research, Guangdong Provincial Key Laboratory of Viral Hepatitis Research, Department of Infectious Diseases, Nanfang Hospital, Southern Medical University, 510515 Guangzhou, Guangdong China; 3 Guangdong Provincial Key Laboratory of Gastroenterology, Department of Gastroenterology, Nanfang Hospital, Southern Medical University, Guangzhou, China; 4 Department of Respiratory Diseases, Nanfang Hospital, Southern Medical University, Guangzhou, China

**Keywords:** Bacterial infection, Cell death

## Abstract

Extracellular vesicles (EVs) have emerged as important vectors of intercellular dialogue. High mobility group box protein 1 (HMGB1) is a typical damage-associated molecular pattern (DAMP) molecule, which is cytotoxic and leads to cell death and tissue injury. Whether EVs are involved in the release of HMGB1 in lipopolysaccharide (LPS)-induced acute liver injuries need more investigation. EVs were identified by transmission electron microscopy, nanoparticle tracking analysis (NTA), and western blotting. The co-localization of HMGB1, RAGE (receptor for advanced glycation end-products), EEA1, Rab5, Rab7, Lamp1 and transferrin were detected by confocal microscopy. The interaction of HMGB1 and RAGE were investigated by co-immunoprecipitation. EVs were labeled with the PKH67 and used for uptake experiments. The pyroptotic cell death was determined by FLICA 660-YVAD-FMK. The expression of NLRP3 (NOD-like receptor family pyrin domain containing 3) inflammasomes were analyzed by western-blot or immunohistochemistry. Serum HMGB1, ALT (alanine aminotransferase), AST (aspartate aminotransferase), LDH (lactate dehydrogenase) and MPO (myeloperoxidase) were measured using a commercial kit. The extracellular vesicle HMGB1 was detected in the serums of sepsis patients. Macrophages were found to contribute to HMGB1 release through the EVs. HMGB1-RAGE interactions participated in the loading of HMGB1 into the EVs. These EVs shuttled HMGB1 to target cells by transferrin-mediated endocytosis leading to hepatocyte pyroptosis by the activation of NLRP3 inflammasomes. Moreover, a positive correlation was verified between the sepsis serum EVs-HMGB1 level and clinical liver damage. This finding provides insights for the development of novel diagnostic and therapeutic strategies for acute liver injuries.

## Key points

- HMGB1 is released by serum EVs in sepsis patients.
- Macrophages contribute to the EVs-HMGB1 release.
- LPS induces the sorting of HMGB1 into EVs which depend on RAGE.
- Macrophage-derived EVs shuttle HMGB1 to hepatocytes by transferrin-mediated endocytosis and subsequently promote hepatocyte pyroptosis by activating the NLRP3 inflammasome.

## Introduction

Extracellular vesicles (EVs) have emerged as important vectors of intercellular communication [1, 2]. There is evidence that EVs have crucial roles in many liver diseases including hepatitis viral infections, hepatocellular carcinoma, liver fibrosis, non-alcoholic fatty liver disease, alcoholic liver disease, and liver-specific tumor metastasis [3, 4]. However, the role of EVs in sepsis-induced acute liver injuries remains unclear and requires further investigation.

Damage associated molecular pattern (DAMP) molecules have key roles in the progression of lipopolysaccharide (LPS)-induced acute liver injuries. HMGB1 is a typical DAMP molecule that is cytotoxic and leads to cell death and tissue injury [5]. HMGB1 release occurs through two major pathways: passive release and active release. Active secretion always requires two steps: the translocation of nuclear HMGB1 to the cytoplasm and the gradual induction of programmed, pro-inflammatory cell death that enables cytoplasmic HMGB1 to reach the extracellular space [6]. One study has shown that amnion epithelial cells release exosomes containing HMGB1 to other uterine tissues to produce parturition-associated inflammatory changes [7]. Remarkably, the neutralization of inflammation by HMGB1-specific antibodies prevents endotoxin-induced lethality [8, 9]. It is unknown whether EVs are involved in the release of HMGB1 in LPS-induced acute liver injuries.

LPS has a pivotal role in the pathogenesis of acute liver injuries. Kupffer cells (KCs), resident hepatic macrophages, are well characterized in response to LPS [10]. Through toll-like receptor 4 (TLR4), KCs take up endotoxins and contribute significantly to inflammation and liver damage [10]. It remains unclear whether macrophage-derived EVs contain HMGB1. NLRP3 inflammasomes are mediators of LPS-induced acute liver injury [11]. NLRP3 activation causes the activation of caspase-1, which cleaves pro-IL-1β and pro-IL-18 to their mature forms and induces cell pyroptosis [12]. HMGB1 has a strong correlation with NLRP3 inflammasome activation. A previous study has shown that heatstroke-induced HMGB1 binds to the receptor for advanced glycation end-products (RAGE), leading to NLRP3 inflammasome activation and subsequent pyroptosis of hepatocytes [13]. Xu et al. revealed that HMGB1 initiates HMGB1 endocytosis through RAGE binding, which in turn induces cell pyroptosis [14]. A recent article has shown that in lethal sepsis, HMGB1 can mediate caspase-11-induced pyroptosis [15]. It is unclear whether HMGB1 in EVs activates NLRP3 in recipient cells and causes pyroptosis.

Consequently, we hypothesized that LPS induces the release of HMGB1-loaded EVs from macrophages to trigger the pyroptosis of hepatocytes through the NLRP3 inflammasome pathway. This study provides further insights into the importance of macrophage-derived EVs in LPS-induced acute liver injury.

## Results

### Macrophages contribute to the HMGB1 release by EVs

**Fig. 1:**
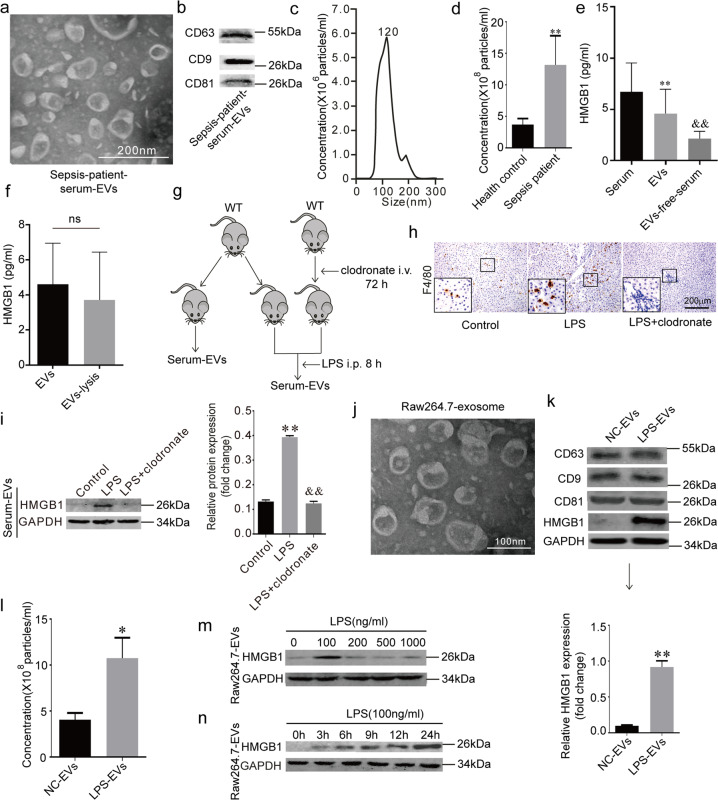
Macrophages contribute to the HMGB1 release by EVs. **a** TEM (transmission electron microscopy) of serum-derived EVs (extracellular vesicles) from sepsis patients. Scale bar = 200 nm. **b** Biomarkers of sepsis patient serum EVs. Immunoblots are representative of three separate experiments. **c**, **d** Size distribution and number of EVs analyzed by NTA. **e** Serum-HMGB1, EVs-HMGB1 and EVs-free-serum HMGB1 levels in the sepsis patients. (**) *P* < 0.01 versus Serum group; (^&&^) *P* < 0.01 versus EVs group. **f** EVs HMGB1 and EVs-lysis HMGB1 levels in the sepsis serum, ns: no significance. **g** Macrophages were cleaned out with clodronate for 48 h and exposed to LPS for another 8 h, serum-derived EVs were collected. **h** F4/80 staining in all groups; Scale bar = 200 μm. **i** HMGB1 level in serum-derived EVs in all groups, the proteins were normalized by GAPDH, (**) *P* < 0.01 versus control-EVs group; (^&&^) *P* < 0.01 versus LPS-EVs group. **j** TEM of Raw264.7 EVs. Scale bar = 100 nm. **k** EVs biomarkers and HMGB1 in Raw264.7 derived EVs. Immunoblots are representative of three separate experiments. **l** Number of EVs analyzed by NTA. **m** HMGB1 in Raw264.7 derived EVs exposed to different concentrations of LPS protein was normalized to GAPDH protein. Immunoblots are representative of three separate experiments. **n** EVs HMGB1 and levels in the LPS-induced macrophage culture medium at different time points. (*) *P* < 0.05 versus 0 h group; (**) *P* < 0.01 versus 0 h group.

We isolated serum EVs (Fig. 1a) from sepsis patients (patient information is shown in Table 1). The common markers of the EVs (*CD63*, *CD9*, and *CD81*) were detected with western blotting (Fig. 1b). Nanoparticle tracking analysis revealed that the diameters of the EVs were approximately 120 nm (Fig. 1c). The counts for the serum EVs in the sepsis patients were higher than that of the healthy controls (Fig. 1d). We divided the serums of sepsis patients into three parts: serum, EVs, and EVs-free serum. HMGB1 was detected by ELISA, and it was found that the EVs contained more HMGB1 than that of the EVs-free serum (Fig. 1e). It is possible that HMGB1 within the EVs could not be detected by ELISA. We conducted further analyses to detect HMGB1 after lysis of the EVs and found no significant difference in the amount of HMGB1 between the EVs and EVs-lysis groups (Fig. 1f). The above results show that HMGB1 in the peripheral blood serum of the sepsis patients may exist in the form of EVs.

**Table 1:** Characteristics of health controls and sepsis patients enrolled.

Group	Health control	Sepsis patients
Number	8	31
Gender (male/female)	4/4	16/15
Age(years)	48.40 ± 5.18	56.20 ± 14.40
ALT (U/L) (Mean±SD)	17.12 ± 9.23	91.38 ± 70.27
AST (U/L) (Mean±SD)	18.00 ± 4.27	73.06 ± 49.34
CRP (mg/L) (Mean±SD)	1.92 ± 0.83	55.56 ± 29.32
Pct (mg/L) (Mean±SD)	0.018 ± 0.011	17.18 ± 21.74
DBIL (μmol/L) (Mean±SD)	4.85 ± 1.62	20.43 ± 8.22
IBIL (μmol/L) (Mean±SD)	7.49 ± 2.91	8.76 ± 6.52
TBIL (μmol /L) (Mean±SD)	12.35 ± 3.21	29.20 ± 9.57

Macrophages can release HMGB1 into the extracellular space, therefore leading to cell death and tissue injury [5]. We hypothesized that macrophages might contribute to HMGB1 release through the EVs. We cleaned out mice macrophages with clodronate and exposed the mice to LPS (Fig. 1g). The F4/80 staining confirmed that the macrophages were cleaned out (Fig. 1h) in the liver. By measuring the number of HMGB1^+^ serum-EVs (EVs loaded with HMGB1), we found that the clodronate decreased the number of HMGB1^+^ serum-EVs compared to the LPS group (Fig. 1i). Next, we isolated EVs from Raw264.7 cells. TEM was performed to reveal the structure of the EVs (Fig. 1j). The common markers (*CD63*, *CD9*, and *CD81*) for EVs and HMGB1 were detected using western blotting (Fig. 1k). LPS increased the number of released Raw264.7 EVs compared to the control group (Fig. 1l). Interestingly, we found that LPS also increased the level of HMGB1 within the EVs derived from the Raw264.7 cells (Fig. 1m–n), and 100 ng/ml of LPS induced the highest expression of HMGB1^+^ EVs (Fig. 1m). Moreover, HMGB1 was expressed at normal levels within the nuclei while LPS induced the nucleocytoplasmic translocation of HMGB1 (Fig. S1). These results show that macrophages contribute to the release of HMGB1 by the EVs.

### LPS-induced macrophage EVs cause liver damage

**Fig. 2:**
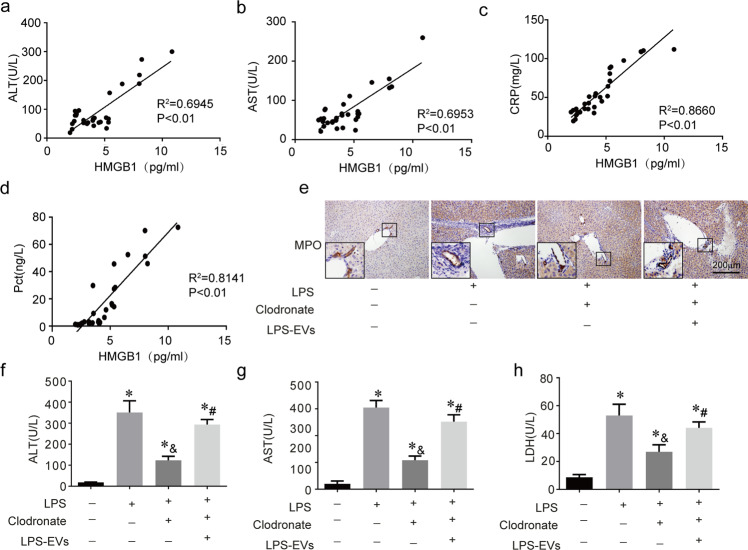
LPS-induced macrophage EVs cause liver damage. **a**–**d** The EVs HMGB1 in sepsis patients serum were correlated with the levels of ALT (alanine aminotransferase), AST (aspartate aminotransferase), CRP (C-reactive protein), and Pct (procalcitonin). *n* = 5 mice per group. **e** MPO (myeloperoxidase) staining in the liver of all groups; Scale bar = 200 μm. **f**–**h** Serum ALT, AST and LDH (lactate dehydrogenase) levels in all groups. *n* = 5 mice per group. (*) *P* < 0.01 versus control group; (^&^) *P* < 0.01 versus LPS group; (^#^) *P* < 0.01 versus clodronate group.

From the clinical data, we found that the level of HMGB1^+^ serum-EVs was positively correlated with the level of liver injury in the patient (Fig. 2a–b) and the inflammation index (C-reactive protein (CRP) and procalcitonin (PCT)) (Fig. 2c–d). We labeled macrophage EVs with PKH67 and injected the cocktail into mice. We found that the main absorption organ was the liver (Fig. S2). After cleaning out the macrophages with clodronate for 48 h, we injected LPS together with LPS-induced Raw264.7 EVs into the mice for another 8 h. The inflammatory response (myeloperoxidase (MPO) expression) decreased when the macrophages were cleaned out with clodronate when exposed to LPS (Fig. 2e). Interestingly, the inflammatory response was rescued by the LPS-induced macrophage EVs when the mice macrophages were cleaned out. The ALT, AST, and LDH levels showed the same trends (Fig. 1f–h). These results suggest that LPS-induced macrophage EVs cause liver damage.

### HMGB1 absorbed into the EVs is depend on cytoplasmic RAGE

**Fig. 3:**
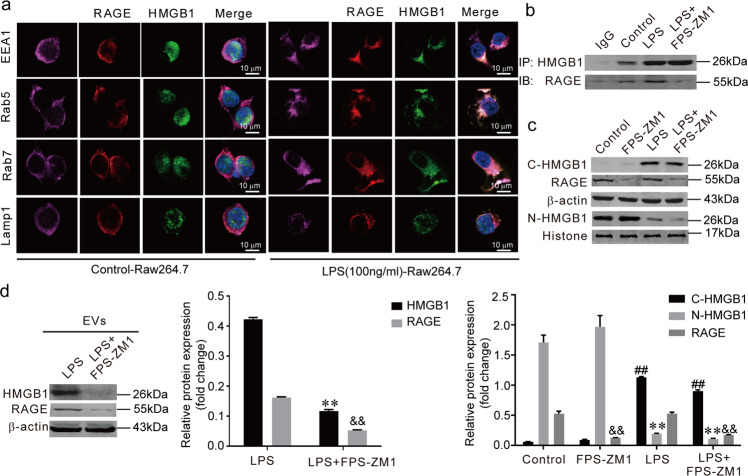
HMGB1 absorbed into the EVs is depend on cytoplasmic RAGE. **a** The co-localization of cytoplasmic HMGB1, RAGE, endosome markers (EEA1, Rab5, Rab7) and lysosomal marker Lamp1. **b** The interaction between HMGB1 and RAGE was measured through Co-IP (co-immunoprecipitation). **c** The effect of FPS-ZM1 on the nucleocytoplasmic translocation of HMGB1. (**) *P* < 0.01 versus control group; (^&&^) *P* < 0.01 versus control group; (^##^) *P* < 0.01 versus control group. The protein levels were normalized to GAPDH and Histone 3.1. **d** The protein levels of HMGB1 an**d** RAGE within EVs. (**) *P* < 0.01 versus LPS group. (^&&^) *P* < 0.01 versus LPS group. The results shown are representative of three independent experiments.

Next, we investigated how the HMGB1 was loaded into the EVs and released into the extracellular space. First, we observed that the RAGE was co-localized with the endosome system (EEA1, Rab5, and Rab7). When LPS-induced HMGB1 translocated from the nucleus to the cytoplasm, cytoplasmic HMGB1 co-localized with the RAGE and endosome system rather than with the lysosomal marker (Lamp1) of the Raw264.7 cells (Fig. 3a). Co-IP (co-immunoprecipitation) analysis further confirmed that the interaction between HMGB1 and RAGE could be blocked with FPS-ZM1 (RAGE inhibitor) (Fig. 3b). Meanwhile, FPS-ZM1 did not affect the nuclear translocation of HMGB1 (Fig. 3c). FPS-ZM1 reduced the protein levels of HMGB1 and RAGE in the EVs (Fig. 3d). Collectively, these findings show that HMGB1 is delivered into the EVs which is depend on cytoplasmic RAGE before being released into the extracellular space.

### EVs shuttle HMGB1 to AML-12 by transferrin-mediated endocytosis

**Fig. 4:**
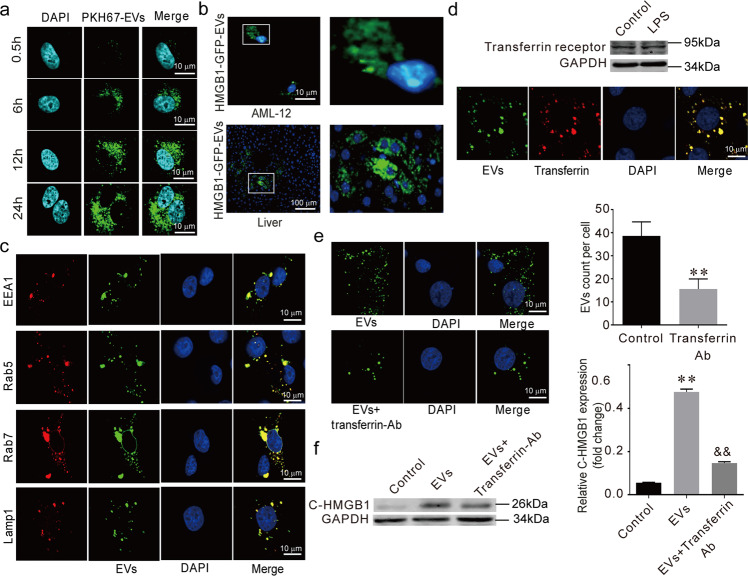
EVs shuttle HMGB1 to AML-12 by transferrin-mediated endocytosis. **a** Representative confocal microscopy micrographs of AML-12 cells after co-culture with purified PKH67-EVs. **b** HMGB1-GFP EVs uptake occurred in vitro and in vivo. **c** The co-localization of internalized EVs and AML-12 endosomes (EEA1, Rab5, Rab7) or Lamp1. **d** The expression of transferrin receptor in EVs, the co-localization of internalized EVs with AML-12 transferrin. **e** The LPS-EVs absorbed by AML-12 cells. **f** Cytoplasmic HMGB1 in AML-12 cells. (**) *P* < 0.01 versus control group. (^##^) *P* < 0.01 versus EVs group.

Next, we chose AML-12 (hepatocyte cell line) as the recipient cells of the macrophage-released EVs. With the transwell system, we labeled the EVs with PKH67 (green) and added these EVs into the AML-12 cells. Using confocal microscopy, we found that the AML-12 cells absorbed the Raw264.7-derived EVs (Fig. 4a). We collected GFP-labeled-HMGB1^+^ EVs from the LV-HMGB1-GFP-Raw264.7 cell line. The AML-12 cells and liver absorbed the GFP-labeled-HMGB1^+^ EVs (Fig. 4b).

After internalization in the hepatocytes, we found that the EVs co-localized with the endosomal/lysosomal EEA1, Rab5, Rab7, and Lamp1 (Fig. 4c). Interestingly, we found that the EVs expressed transferrin receptors (Fig. 4d) and that the absorbed EVs were co-localized with AML-12 transferrin. Furthermore, the uptake rate of the EVs and cytoplasmic HMGB1 in the target cells decreased significantly in response to transferrin neutralizing antibody (Fig. 4e, f). These results show that EVs secreted from Raw264.7 cells shuttled HMGB1 to AML-12 cells through transferrin-mediated endocytosis.

### LPS-EVs promote the pyroptosis of hepatocytes

**Fig. 5:**
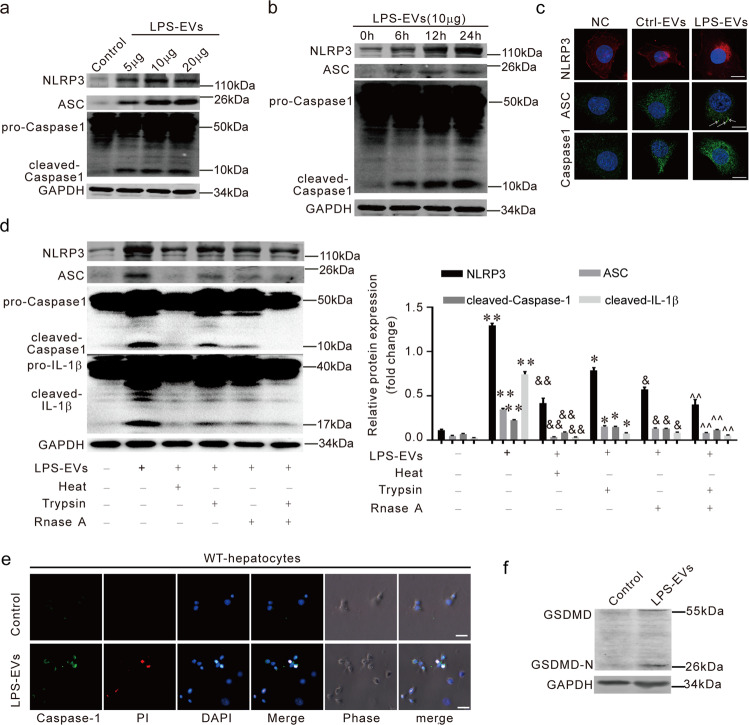
LPS-EVs promote the pyroptosis of hepatocytes. **a**–**c** AML-12 cells were treated with LPS-EVs. NLRP3, ASC, and Caspase-1 protein levels were measured by immunoblotting and in confocal micrographs. (*) *P* < 0.05 versus control group; (^&&^) *P* < 0.01 versus control group; (^##^) *P* < 0.01 versus control group. **d** The NLRP3 inflammasome activation was analyzed when AML-12 cells were challenged with DMEM, LPS-EVs, boiled EVs (100 °C, 10 min), RNase A treated EVs, Trypsin-treated EVs or (RNase A + Trypsin)-treated EVs. **e** AML-12 pyroptosis was measured by immunofluorescence. **f** The GSDMD expression in LPS-exosomes treated AML-12.

To find the function of LPS-EVs, we focused on the biological effects of the LPS-EVs on hepatocytes because we already found that LPS-induced macrophage EVs cause liver damage. We found that LPS-EVs activated the NLRP3 inflammasomes in AML-12 cells in a dose- and time-dependent manner (Fig. 5a, b). Immunofluorescence also showed that the NLRP3 inflammasomes were induced by LPS-EVs (Fig. 5c). When the protein or RNA was removed from the EVs, LPS-EVs induced NLRP3 inflammasome activation was blocked (Fig. 5d). This shows that the proteins and RNAs within the LPS-induced macrophage EVs were both involved in the activation of the hepatocyte NLRP3 inflammasomes. From the immunofluorescence, we found that LPS-EVs induced pyroptosis in the AML-12 cells (Fig. 5e). Recent studies have identified pyroptosis as gasdermin-mediated programmed necrotic cell death. Gasdermin D (GSDMD) is a substrate for both caspase-1 and caspase-11/4/5 [16]. We detected GSDMD-N domains in LPS EVs-treated AML-12 cells, which shows that these EVs triggered the pyroptosis in the AML-12 cells (Fig. 5f). These results suggest that LPS EVs activate NLRP3 inflammasome in hepatocytes and cause the pyroptosis of hepatocytes.

### HMGB1^+^ EVs inducing hepatocyte pyroptosis and activate NLRP3 inflammasomes

**Fig. 6:**
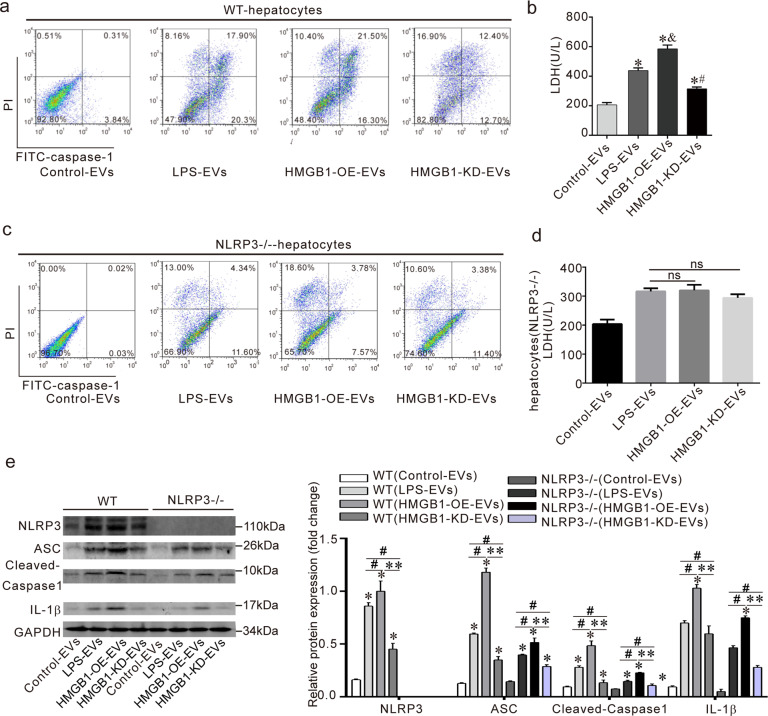
HMGB1^+^ EVs activate NLRP3 inflammasomes inducing hepatocyte pyroptosis. **a**, **c** The primary hepatocytes were isolated from WT and NLRP3^−/−^ mice, the percentage of pyroptotic primary hepatocytes from WT and NLRP3^−/−^ mice were measured by flow cytometry. **b**, **d** The level of LDH was examined in wild-type and NLRP3^−/−^ primary hepatocytes when treated with different EVs. (*) *P* < 0.05 versus control medium; (^&^) *P* < 0.05 versus control LPS-EVs; (^#^) *P* < 0.05 versus LPS^-^EVs. **e** The activation of the NLRP3 inflammasome was analyzed in wild-type and NLRP3^−/−^ primary hepatocytes when treated with different EVs. Data are expressed as means ± sd. *for *p* < 0.05 vs control group. **for *p* < 0.01; ^##^ for *p* < 0.01.

Whether HMGB1^+^ EVs induced pyroptosis of hepatocytes was not investigated. We isolated primary hepatocytes from wild-type and NLRP3^−/−^ mice. They were then treated with different EVs (control, LPS, HMGB1-OE, and HMGB1-KD). The number of pyroptotic primary hepatocytes caused by HMGB1-overexpressing EVs increased significantly compared to the LPS-EVs group in the wild-type mice. However, the number of pyroptotic primary hepatocyte cells decreased in the HMGB1-knockdown EVs group compared to the LPS-EVs group (Fig. 6a). The released LDH levels of AML-12 showed a similar trend (Fig. 6b).

An interesting thing was that pyroptosis in the NLRP3^−/−^ hepatocytes decreased compared to the wild-type hepatocytes. There was no significant change in the NLRP3^−/−^ hepatocytes pyroptosis and LDH among the HMGB1-OE-EVs group and the LPS EVs or the HMGB1-KD-EVs group and the LPS EVs (Fig. 6c, d). These results show that the pyroptosis of the hepatocytes caused by the HMGB1^+^ EVs depended on NLRP3 activation in the cells.

For the role of HMGB1 in LPS-EVs toward hepatocytes NLRP3 activation. Our results show that HMGB1-overexpressing EVs promoted the activation of the NLRP3 inflammasome/IL-1β, while HMGB1-knockdown EVs decreased the activation of the NLRP3 inflammasomes/IL-1β axis compared to LPS EVs (Fig. 6e). When we knocked out the NLRP3 in hepatocytes, the activation of the NLRP3 inflammasome/IL-1β axis in the NLRP3^−/−^ primary hepatocytes decreased compared to that of the wild-type primary hepatocytes (Fig. 6e), suggesting that HMGB1 in the EVs activated the NLRP3 inflammasomes in the target cells.

### HMGB1^+^ EVs cause liver damage and activate NLRP3 inflammasomes in the liver

**Fig. 7:**
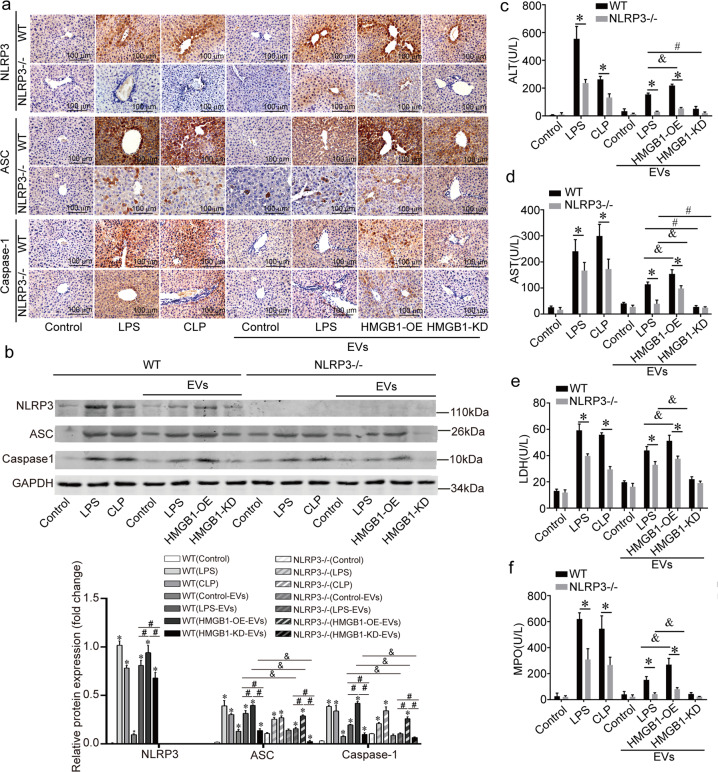
HMGB1^+^ EVs cause liver damage and activate NLRP3 inflammasomes in the liver. **a** EVs were collected from Raw264.7 (control, HMGB1-Overexpressing and HMGB1-Knockdown) were injected into WT and NLRP3^−/−^mice. The LPS or CLP surgery models served as the sepsis positive models. Expression of NLRP3, ASC and Caspase-1 was evaluated; scale bar = 100 μm. *n* = 5 mice per group. **b** The expression levels of NLRP3/ASC/Caspase-1 were analyzed by immunoblotting. Data are expressed as means ± sd. * for *p* < 0.01 vs control group. ^#^ for *p* < 0^.^05; ^&^ for *p* < 0.05. **c**–**f** Serum ALT, AST, LDH and MPO levels. Data are expressed as means ± sd. * for *p* < 0.01. ^&^ for *p* < 0.05; ^#^ for *p* < 0.01. *n* = 5 mice per group.

We further focused on the effects of the HMGB1^+^ EVs in vivo. Raw264.7-derived EVs (control, HMGB1-overexpressing, and HMGB1-knockdown) were injected into wild-type mice and NLRP3^−/−^ mice through the tail vein. The results showed that the NLRP3/ASC/caspase-1 level increased in the LPS, CLP (cecal ligation puncture), LPS EVs, and HMGB1-overexpressing EVs groups. However, when HMGB1^+^ EVs were knocked down, the NLRP3/ASC/caspase-1 level decreased significantly (Fig. 7a–b). We also found that the expression of ASC and cleaved-caspase-1 decreased in all the groups of NLRP3^−/−^ mice compared to the WT mice (Fig. 7a–b). The serum analysis of the ALT, AST, LDH, and MPO levels showed the same trends (Fig. 7c–f).

### Serum-derived EVs of patients with sepsis express high HMGB1 and cause liver injury in vivo

**Fig. 8:**
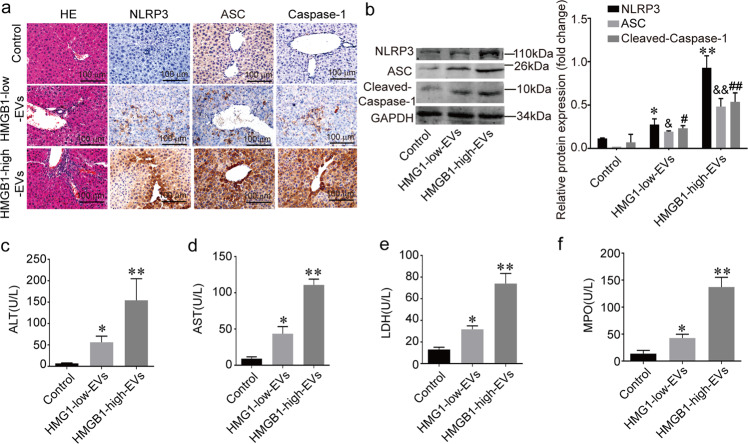
Serum-derived EVs of patients with sepsis express high HMGB1 and cause liver injury in vivo. **a** HE and IHC staining of MPO, F4/80, NLRP3, ASC, and Caspase-1 in all groups. n = 5 mice per group. **b** The NLRP3 inflammasome was detected by immunoblotting. **c**–**f** The ALT, AST, LDH, and MPO levels. (*) *P* < 0.05 versus control group. (^&^) *P* < 0.05 versus control group; (^#^) *P* < 0.05 versus control group; (**) *P* < 0.01 versus control group. (^&&^) *P* < 0.01 versus control group; (^##^) *P* < 0.01 versus control group. *n* = 5 mice per group.

To deduce the function of serum HMGB1^+^ EVs in sepsis patients, we injected HMGB1-low serum EVs and HMGB1-high serum EVs into wild-type mice. Additionally, other components were different in these two types of EVs. We still found NLRP3 inflammasome activation in the HMGB1-high serum EVs group compared to the HMGB1-low serum EVs group (Fig. 8a, b). The serum analysis showed that the ALT, AST, LDH, and MPO levels in the HMGB1-high serum EVs increased compared to the HMGB1-low serum EVs (Fig. 8c–f). These results show that the sepsis serum-derived EVs express a high level of HMGB1, which causes liver injury and NLRP3 inflammasome activation.

## Discussion

The present study demonstrates that LPS-induced HMGB1^+^ EVs from macrophages trigger hepatocyte pyroptosis by activating the NLRP3 inflammasome. The main findings include the following: (1) HMGB1 released by EVs in the serum and macrophages contribute to EVs-HMGB1 release; (2) LPS induces the sorting of HMGB1 into EVs which depend on RAGE; () Macrophage-derived EVs shuttle HMGB1 to hepatocytes by transferrin-mediated endocytosis and subsequently promote hepatocyte pyroptosis by activating the NLRP3 inflammasome.

We report that LPS induces macrophages to release HMGB1 as the cargo of the EVs. Compared to early inflammatory mediators (such as TNF-α, IL-6, and IL-1β), which have limited clinical significance due to their narrow therapeutic window, HMGB1 has a prolonged therapeutic window as a late mediator in lethal systemic inflammation [17]. Circulating TNF-α and IL-6 are elevated for 5 days after the onset of sepsis, and serum HMGB1 levels are elevated from day 7 until at least day 28 [17]. Interestingly, our data showed that EVs in patients with sepsis are important carriers of HMGB1 than free-state HMGB1, and the lysis of EVs or not has no significant effect on the ELISA detection of HMGB1. Due to the stability of the EVs and the development of detection methods, HMGB1^+^ EVs can be a promising biomarker for sepsis diagnosis. Herein, we found that HMGB1-loaded EVs could be secreted from macrophages at the very beginning of cell injury.

The mechanisms behind the cargo loading of EVs are still poorly understood. Studies have revealed that the ESCRT machinery is essential for controlling specific protein sorting into intraluminal vesicles (ILVs) within the first step of ubiquitination [17, 18]. Kang, R [6]. and Yiting Tang [17] et al. have shown that cytoplasmic HMGB1 is modified by posttranscriptional modifications of hyperacetylation instead of ubiquitination after HMGB1 localizes from the nucleus to the cytoplasm. Our results confirm that cytoplasmic HMGB1 is transferred into multivesicular bodies (MVB) by binding to RAGE expressed in the endosome system. We show that RAGE has a vital role during the sorting of HMGB1 into the EVs. Our study suggests that the receptor–ligand pattern is an important mechanism during the cargo sorting of EVs.

Previous reports have revealed that the binding and uptake of EVs involve many pathways [1, 2]. The selective uptake of EVs may be critical for them to function properly. For instance, exosomes engineered to express the EGFR ligand on their surfaces preferentially bind to EGFR-expressing breast cancer cells and successfully deliver microRNA to recipient cells [19]. Our results show the participation of the transferrin pathway in the uptake of EVs by hepatocytes. When we blocked transferrin in the hepatocytes with a neutralizing antibody, the percentage of internalized EVs decreased dramatically. Our study thus suggests that the interaction between transferrin and its receptor has a role in the uptake of macrophage-derived EVs by hepatocytes.

Wree [20]. et al. reported that continuous NLRP3 inflammasome activation induces hepatocyte pyroptosis and subsequent severe liver damage in mice. Our data suggest a direct effect of HMGB1 + EVs on hepatocyte pyroptosis. Previous studies have highlighted the function of HMGB1 through the binding of receptors to activate pro-inflammatory signaling [6]. Ben Lu et al. discovered that hepatocyte-released high mobility group box 1 (HMGB1) was required for caspase-11-dependent pyroptosis and lethality in endotoxemia and bacterial sepsis. Mechanistically, hepatocyte-released HMGB1 bound LPS and targeted its internalization into the lysosomes of macrophages and endothelial cells via the receptor for advanced glycation end-products (RAGE) [21]. However, our results show that HMGB1^+^ EVs can induce pyroptosis once absorbed by recipient cells. This function may be responsible for the occurrence of inflammation in LPS-induced acute liver injuries.

From the clinical results, we propose that serum HMGB1 + EVs in sepsis patients can be used as a marker for sepsis diagnosis. Previous research has shown that HMGB1 appears to be an early stage mediator in trauma [22], while HMGB1 is conversely a late-stage mediator in sepsis [23]. Wang showed that treatment of FeTPPS (a small molecule selectively inhibits HMGB1- mediated caspase-11 activation) attenuates HMGB1- and caspase-11-mediated immune responses, organ damage, and lethality in endotoxemia and bacterial sepsis [24]. Li W report that the exosome was an important pathway for HMGB1 release from hepatocytes; which was dependent on TLR4 [25]. Here, we found that the serum-derived EVs of sepsis patients and macrophages contain high levels of HMGB1 and stimulate the activation of the NLRP3 inflammasome and liver damage. It indicated that both macrophages and hepatocytes could release HMGB1through extracellular vesicles.

Numerous animal studies have revealed that HMGB1 is a negative outcome predictor of sepsis [26]. In humans, HMGB1 circulatory levels persistently increase in severe sepsis and septic shock [23, 26]. This increase seems to be associated with a poor patient outcome or organ failure. Nevertheless, there is no significant difference in the survival rate [27]. In fact, patients who survive severe sepsis maintain a continuously high HMGB1 circulatory level [27]. Our findings suggest that HMGB1 has the potential as a diagnostic marker for sepsis.

This study had several limitations: (1) These Nlrp3 and other components could also have been included in the macrophage-released EVs, which caused activation of NLRP3 inflammasome; (2) We did not explore whether the HMGB1^+^ EVs level could be used as a prognostic marker for sepsis; (3) The released EVs might contain LPS itself which cause a similar effect on hepatocyte, which need further investigation.

In summary, we found that LPS induces HMGB1-loaded EVs release from macrophages by binding to RAGE and that these EVs are internalized by hepatocytes by transferrin-mediated endocytosis, which triggers the pyroptosis of the hepatocytes by activating the NLRP3 inflammasome. This study provides novel insights into possible future diagnostic and therapeutic strategies for acute liver injury.

## Materials and Methods

### Reagents

LPS was provided by Sigma-Aldrich (Sigma-Aldrich, St. Louis, MO), FPS-ZM1 was purchased from selleckchem (Houston, TX, USA), clodronate was purchased from Vrije Universiteit Amsterdam, tryspin and Rnase A were provided by Life Technologies (Invitrogen Life Technologies Inc., CA, USA).

### Human studies

Human studies were approved by the Nanfang hospital, Southern Medical College (Institution Review Board No. 0604008488). Human serum samples were obtained from control healthy subjects and sepsis patients with liver injury at Department of Emergency Medicine and Department of Infectious Diseases (Nanfang Hospital, Southern Medical University, China). Human studies were approved by the Nanfang hospital, Southern Medical College (Institution Review Board No. 0604008488). All individuals provided informed consent for serum donation on approved institutional protocols.

### Animals

Male Wide Type C57BL/6, NLRP3 knock-out mice (NLRP3^−/−^, Jackson laboratory, stock 021302) (6–8 week) were maintained in a specific pathogen-free facility (Central Animal Care Facility of Southern Medical University, Guangzhou, China). Animal experimental procedures were in accordance with the National Institutes of Health guidelines and were approved by the local Animal Care and Use Committee of Southern Medical University.

### Cell culture

The primary hepatocytes were isolated and cultured in RPMI-1640 medium as indicated [28]. Raw264.7 and AML-12 cells obtained from ATCC were cultured in DMEM (GIBCO BRL, Life Technologies, Inc., C11995500BT) with 10% FBS (GIBCO BRL, Life Technologies, Inc.10270-106), penicillin/streptomycin (100 U/ml: 100 μg/ml; Sigma-Aldrich, catalog no. 15140-122) at 37 °C in 5% CO_2_. After overnight incubation, fresh serum-free DMEM medium was added and incubate for 24 h then the medium was collected for exosomes isolation.

### Acute liver injury models

A single dose of LPS (10 mg/kg; Sigma-aldrich, St. Louis, MO) was administered to mice intraperitoneally and CLP were used as sepsis animal models. Mice were anesthetized with 5% chloral hydrate, after which a 2-cm midline laparotomy was performed under aseptic conditions to expose the cecum. A single through-and-through puncture was performed by a 21-gauge needle between the ligation site and the end of the cecum, and then fecal material was extruded through the puncture. The cecum was repositioned into the peritoneal cavity, and the laparotomy was closed. For macrophage deletion, clodronate (200 μl/mice) was injected into mice intraperitoneally for 48 h. For EVs injection, EVs (40 μg) were injected into mice once through tail vein. All mice in this study were randomly divided into different groups. No blinding was done in this research.

### Isolation and identification of extracellular vesicles

Raw264.7-EVs and serum-EVs were isolated and purified as described previously, with added modifications [29]. Briefly, Raw264.7 culture medium and serum were harvested and centrifuged at 300 *g* for 10 min, 2000 *g* for 20 min and 10,000 *g* for 30 min at 4 °C. Then supernatant was filtered through 0.22 μm filters (Millipore, MA, USA, SLGP033RB) to remove contaminating apoptotic bodies, microvesicles and cell debris. Clarified culture medium was then centrifuged in a Beckman Coulter Optima TM L-80XP Ultracentrifuge at 100,000 *g* at 4 °C for 70 min to pellet exosomes. The supernatant was carefully removed, and EVs-containing pellets were resuspended in1 mL of ice-cold PBS and pooled. A second round of ultracentrifugation [100,000 *g* at 4 °C for 70 min with a Type 50.2 Ti rotor (k-factor: 157.7)] was carried out, and the resulting EVs pellet resuspended in 100 μL of PBS and stored at −80 °C. The protein content of the concentrated EVs was determined using a BCA protein assay kit. Raw264.7-EVs were identified by transmission electron microscopy and NTA (NS3000, Worcestershire, UK), tetraspan molecules CD63 (ab10895, Abcam, Cambridge, UK), CD9 (ab92726, Abcam, Cambridge, UK), CD81 (18250-1-AP, Proteintech, Rosemont, USA) were verified by western blotting.

### Confocal microscopy

Raw264.7 or AML-12 cells were fixed with 4% paraformaldehyde and then permeabilized with 0.1% Triton X-100 for 20 min. Cells were washed with PBS, blocked with 3% BSA in Tris buffered saline and then incubated with primary antibodies against HMGB1 (ab18256), RAGE (ab7764), EEA1 (ab70521), Rab5 (ab50523), Rab7 (ab50533), Lamp1 (ab119127), Transferrin (ab82411), NLRP3 (ab4207), ASC (ab47092), Caspase-1 (ab1872) (All antibodies came from Abcam, Cambridge, UK) overnight (at 4 °C at a dilution of 1:200). Cells were washed with PBS and incubated with secondary goat anti-mouse antibody (conjugated to cy3), goat anti-rabbit antibody (conjugated to Alexa Flour 488), donkey anti-goat antibody (conjugated to Alexa Flour 647), respectively (Life Technologies, Carlsbad, CA) at a dilution of 1:500 for 1 h at room temperature. The cells were washed with PBS and images were acquired using with Zeiss LSM 700 laser scanning confocal microscope and were calculated with ZEN software.

### Co-immunoprecipitation

Raw264.7 were stimulated with 100 ng/ml LPS for 24 h. IP and immunoblotting (IB) were performed, the antibodies for IP was anti-HMGB1; the antibodies for IP was anti-RAGE.

### Western blotting

Equal amounts of EVs pellets (10 μg), cell lysates (15 μg) and tissue protein (20 μg), as determined by BCA protein estimation, were boiled with 5× Loading buffer and run on a 12% SDS-PAGE denaturing gel. The proteins were transferred to PVDF membranes. Blots were blocked with 5% BSA for 1 h at room temperature. Blots were probed with primary antibodies [HMGB1 (ab18256), RAGE (ab7764), NLRP3 (ab214185), ASC (ab47092), Caspase-1 (ab1872), IL-1 β (ab9722) (Abcam, Cambridge, UK), GSDMD (ab209845), Transferrin receptor (10084-2-AP, Proteintech, Rosemont, USA), β-actin (60008-1-Ig, Proteintech, Rosemont, USA), GAPDH (60004-1-Ig, Proteintech, Rosemont, USA), Histone H3.1 (17168-1-AP, Proteintech, Rosemont, USA)] diluted in blocking buffer overnight at 4 °C. Blots were washed 3 times with TBS-T and then incubated with HRP conjugated anti-rabbit or anti-mouse Antibody (Li-cor, Nebraska, USA) diluted in blocking buffer at room temperature for 1 h. Blots were washed thoroughly 3 times with TBS-T and was followed by detection with Odyssey and quantitated using a Molecular Dynamics Densitometer with LANE 1D software.

### Extracellular vesicles labelling and trafficking in vitro and in vivo

For co-culture experiments, AML-12 cells at a density of 2 × 10^5^ cells per ml were cultured in a transwell system with a 0.4-μm porous membrane. For EVs uptake experiments, Raw264.7-derived EVs were labelled with the PKH67 Green Fluorescent Cell Linker Kit (Sigma Aldrich, St. Louis, MO, USA): EVs diluted in PBS were added to 1 ml Diluent C. In parallel, 4 μl PKH67 dye was added to 1 ml Diluent C and incubated with the EVs solution for 4 min. Then 2 ml 0.5% BSA/PBS was added and the labeled EVs were washed at 100,000 *g* for 1 h, the EVs pellet was diluted in 100 μl PBS and used for uptake experiments. The transferrin neutralized antibody (1:100, ab82411, Abcam, Cambridge, UK) was used to block the transferrin in hepatocytes.

### Lentiviral packaging and cell infection

The HMGB1-over-expression (or knock down) lentiviral vector (lenti-HMGB1) and lenti-green fluorescent protein [GFP] (the negative control) were purchased from GenePharma (GenePharma, Shanghai, China). Transfections were performed according to the manufacturer’s instructions. Briefly, all lenti vectors were purified to a titer of 1 × 10^9^ TU/ml. At a Multiplicity of infection (MOI) of 10:1 and 5 μg/ml polybrene in growth medium for 6 h after transfection, the medium was exchanged for fresh DMEM medium. At 72 h after transfection, the cells were passaged for use in further experiments.

### Small interfering RNA (siRNA)

siRNA duplexes targeting HMGB1 and negtive control (NC) were designed and synthesized by RiboBio (Guangzhou, China). Transfection was performed using Lipofectamine RNAiMAX (Invitrogen) according to the manufacturer’s instructions. Briefly, 24 h before transfection, Raw264.7 cells were seeded in 6-well plates at 40% confluency, and transfected with 100 nM HMGB1 siRNA or NC siRNA for 72 h.

### Determination of pyroptotic cell death in vitro

To assess pyroptosis in vitro, we designed the following strategy. Active Caspase 1 was measured in AML-12 cells suspensions with FLICA 660-YVAD-FMK (FLICAR 660 in vitro Active Caspase-1 Detection Kit, Immuno Chemistry Technologie, Bloomington, MN, USA) according to manufactures instructions and with propidium iodide (PI) to mark cells with membrane pores (Life technology, Carlsbad, CA, USA). Cells were then analyzed by flow cytometry FACS CantoII (BD Biosciences, San Jose, CA) and fluorescent images were also obtained by confocal microscopy.

### Immunofluorescence

The liver was frozen in liquid nitrogen and embedded in Tissue-TEK OCT compound (4583, Sakura, Japan). For immunofluorescence staining, the tissues were fixed overnight in formalin. Extensive washing with PBS was performed before incubating the slides with DAPI for nuclear staining. Image was observed using with Zeiss LSM 700 laser scanning confocal microscope and was calculated with the ZEN software.

### Histopathological and Immunohistochemistry

Liver tissues were processed for paraffin embedding and were sectioned into 4-μm sections. The sections were stained with hematoxylin and eosin according to standard protocols. For immunohistochemical staining, the liver slides were incubated with diluted primary antibodies against anti-mouse NLRP3 (ab4207), ASC (ab47092), Caspase-1 (ab1872) (Abcam, Cambridge, UK) according to the manufacturer’s instructions. The primary antibody was detected using the biotin-conjugated anti-rabbit (or anti-goat) immunoglobulin G antibody, and then incubated with streptavidin-biotin. The complex was visualized with the DAB reagent for microscopic examination. Removal of the primary antibody from the procedure provided a negative control.

### Serum measurements

Serum was collected and assayed for ALT (alanine aminotransferase), AST (aspartate aminotransferase), LDH (lactate dehydrogenase) and MPO (myeloperoxidase) were measured using (Nanjing Jiancheng Bioengineering Institute, Nanjing, China) according to the instruction of manufacturer.

### ELISA analysis

The levels of HMGB1 in serum were determined using a commercial ELISA kit specific for human HMGB1 (mlbio, Corporation, Shanghai, China). All experiments were performed in triplicate.

### Statistical analysis

Data are expressed as means ± standard deviation. In statistical analysis of 2 groups, a two-tailed Student’s t test was utilized. In analysis of more than 2 groups, ANOVA analyses were performed. All data were analyzed by SPSS17.0 software. Statistical *P* values < 0.05 were considered significantly.

## Supplementary information

Supplement data

## Data Availability

The data used to support the findings of this study are available from the corresponding author upon request.

## References

[1] Colombo M, Raposo G, Thery C (2014). Biogenesis, secretion, and intercellular interactions of exosomes and other extracellular vesicles. Annu Rev Cell Dev Biol..

[2] van Niel G, D’Angelo G, Raposo G (2018). Shedding light on the cell biology of extracellular vesicles. Nat Rev Mol Cell Biol..

[3] Cai S, Cheng X, Pan X, Li J (2017). Emerging role of exosomes in liver physiology and pathology. Hepatol Res..

[4] Devhare PB, Ray RB (2018). Extracellular vesicles: novel mediator for cell to cell communications in liver pathogenesis. Mol Asp Med..

[5] Bianchi ME, Crippa MP, Manfredi AA, Mezzapelle R, Rovere QP, Venereau E (2017). High-mobility group box 1 protein orchestrates responses to tissue damage via inflammation, innate and adaptive immunity, and tissue repair. Immunol Rev..

[6] Kang R, Chen R, Zhang Q, Hou W, Wu S, Cao L (2014). HMGB1 in health and disease. Mol Asp Med..

[7] Sheller-Miller S, Urrabaz-Garza R, Saade G, Menon R (2017). Damage-associated molecular pattern markers HMGB1 and cell-Free fetal telomere fragments in oxidative-Stressed amnion epithelial cell-derived exosomes. J Reprod Immunol..

[8] Stevens NE, Chapman MJ, Fraser CK, Kuchel TR, Hayball JD, Diener KR (2017). Therapeutic targeting of HMGB1 during experimental sepsis modulates the inflammatory cytokine profile to one associated with improved clinical outcomes. Sci Rep..

[9] Wang H, Ward MF, Sama AE (2014). Targeting HMGB1 in the treatment of sepsis. Expert Opin Ther Targets..

[10] Tsutsui H, Nishiguchi S (2014). Importance of Kupffer cells in the development of acute liver injuries in mice. Int J Mol Sci..

[11] Bawa M, Saraswat VA (2013). Gut-liver axis: role of inflammasomes. J Clin Exp Hepatol..

[12] Vanaja SK, Rathinam VA, Fitzgerald KA (2015). Mechanisms of inflammasome activation: recent advances and novel insights. Trends Cell Biol..

[13] Geng Y, Ma Q, Liu YN, Peng N, Yuan FF, Li XG (2015). Heatstroke induces liver injury via IL-1beta and HMGB1-induced pyroptosis. J Hepatol..

[14] Xu J, Jiang Y, Wang J, Shi X, Liu Q, Liu Z (2014). Macrophage endocytosis of high-mobility group box 1 triggers pyroptosis. Cell Death Differ..

[15] Deng M, Tang Y, Li W, Wang X, Zhang R, Zhang X (2018). The endotoxin delivery protein HMGB1 mediates caspase-11-dependent lethality in sepsis. Immunity..

[16] Shi J, Zhao Y, Wang K, Shi X, Wang Y, Huang H (2015). Cleavage of GSDMD by inflammatory caspases determines pyroptotic cell death. Nature..

[17] Tang Y, Zhao X, Antoine D, Xiao X, Wang H, Andersson U (2016). Regulation of posttranslational modifications of HMGB1 during immune responses. Antioxid Redox Signal..

[18] Raiborg C, Stenmark H (2009). The ESCRT machinery in endosomal sorting of ubiquitylated membrane proteins. Nature..

[19] Ohno S, Takanashi M, Sudo K, Ueda S, Ishikawa A, Matsuyama N (2013). Systemically injected exosomes targeted to EGFR deliver antitumor microRNA to breast cancer cells. Mol Ther..

[20] Wree A, Eguchi A, McGeough MD, Pena CA, Johnson CD, Canbay A (2014). NLRP3 inflammasome activation results in hepatocyte pyroptosis, liver inflammation, and fibrosis in mice. Hepatology..

[21] Deng M, Tang Y, Li W, Wang X, Zhang R, Zhang X (2018). The endotoxin delivery protein HMGB1 mediates caspase-11-dependent lethality in sepsis. Immunity..

[22] Cohen MJ, Brohi K, Calfee CS, Rahn P, Chesebro BB, Christiaans SC (2009). Early release of high mobility group box nuclear protein 1 after severe trauma in humans: role of injury severity and tissue hypoperfusion. Crit Care..

[23] Sunden-Cullberg J, Norrby-Teglund A, Rouhiainen A, Rauvala H, Herman G, Tracey KJ (2005). Persistent elevation of high mobility group box-1 protein (HMGB1) in patients with severe sepsis and septic shock. Crit Care Med..

[24] Wang X, Li Z, Bai Y, Zhang R, Meng R, Chen F (2021). A small molecule binding HMGB1 inhibits caspase-11-mediated lethality in sepsis. Cell Death Dis..

[25] Li W, Deng M, Loughran PA, Yang M, Lin M, Yang C (2020). LPS Induces Active HMGB1 Release From Hepatocytes Into Exosomes Through the Coordinated Activities of TLR4 and Caspase-11/GSDMD Signaling. Front Immunol..

[26] Karlsson S, Pettila V, Tenhunen J, Laru-Sompa R, Hynninen M, Ruokonen E (2008). HMGB1 as a predictor of organ dysfunction and outcome in patients with severe sepsis. Intensive Care Med..

[27] Angus DC, Yang L, Kong L, Kellum JA, Delude RL, Tracey KJ (2007). Circulating high-mobility group box 1 (HMGB1) concentrations are elevated in both uncomplicated pneumonia and pneumonia with severe sepsis. Crit Care Med..

[28] Mohar I, Brempelis KJ, Murray SA, Ebrahimkhani MR, Crispe IN (2015). Isolation of non-parenchymal cells from the mouse liver. Methods Mol Biol..

[29] Coumans F, Brisson AR, Buzas EI, Dignat-George F, Drees E, El-Andaloussi S (2017). Methodological guidelines to study extracellular vesicles. Circ Res..

