# Supplementary material for: LPS-induced macrophage HMGB1-loaded extracellular vesicles trigger hepatocyte pyroptosis by activating the NLRP3 inflammasome
Source: Cell Death Discov. 2021 Nov 6;7:337. doi: 10.1038/s41420-021-00729-0 (PMC8572226; doi:10.1038/s41420-021-00729-0)
Supplement: Supplementary file 1 — Supplement data [file 41420_2021_729_MOESM1_ESM.docx]

**Supplement data**


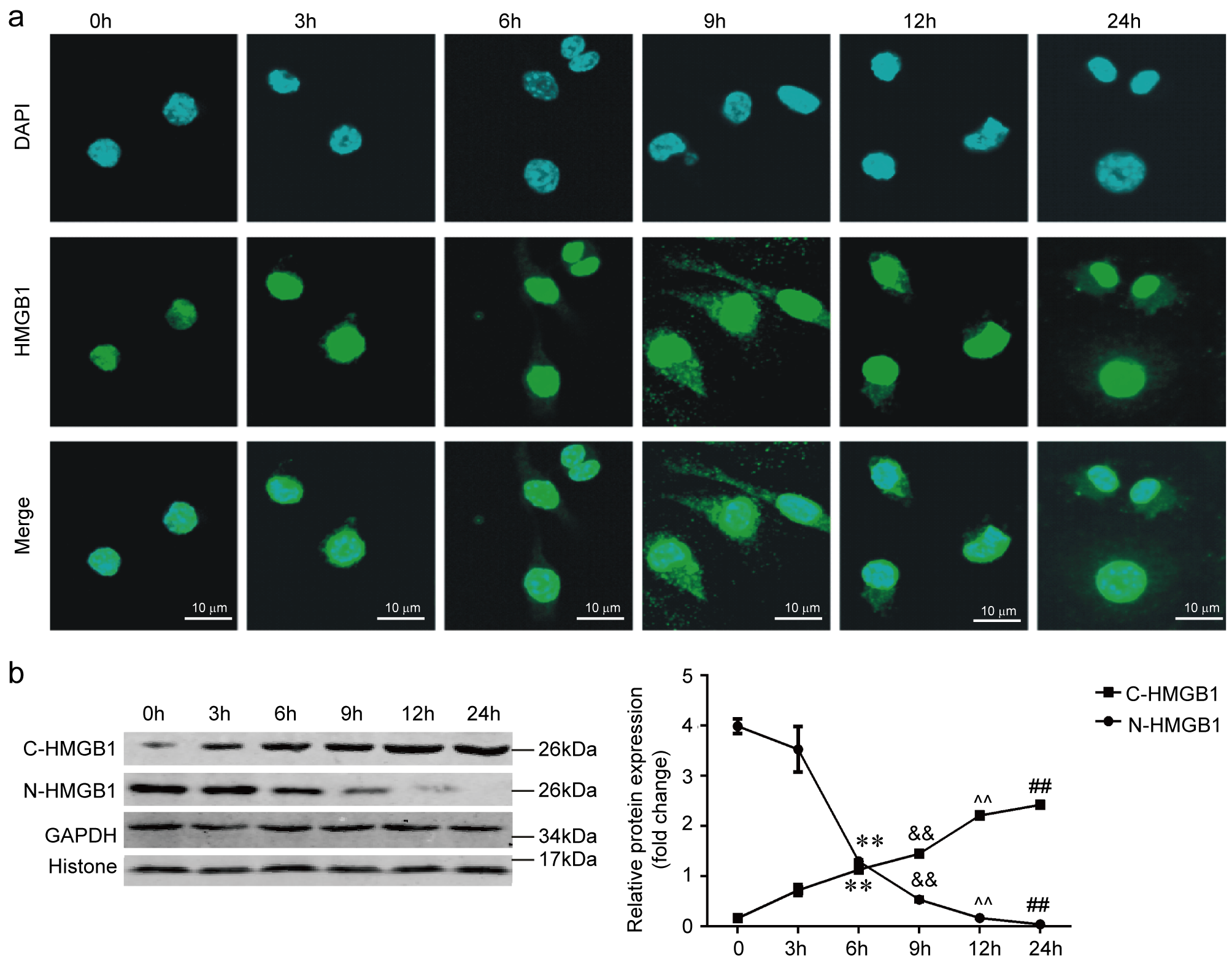


**Figure S1 LPS induced the nucleocytoplasmic translocation of HMGB1. a, b** HMGB1 nucleocytoplasmic translocation was measured by laser scanning confocal microscopy and immunoblot. Nuclear protein was normalized to histone H3.1 protein, and cytoplasmic protein was normalized to GAPDH protein. (*) P < 0.05 versus control group. (**) P < 0.01 versus control group. (&&) P < 0.01 versus control group; (##) P < 0.01 versus control group; (^^) P < 0.01 versus control group; C-HMGB1: cytoplasmic HMGB1; N-HMGB1: nuclear HMGB1.


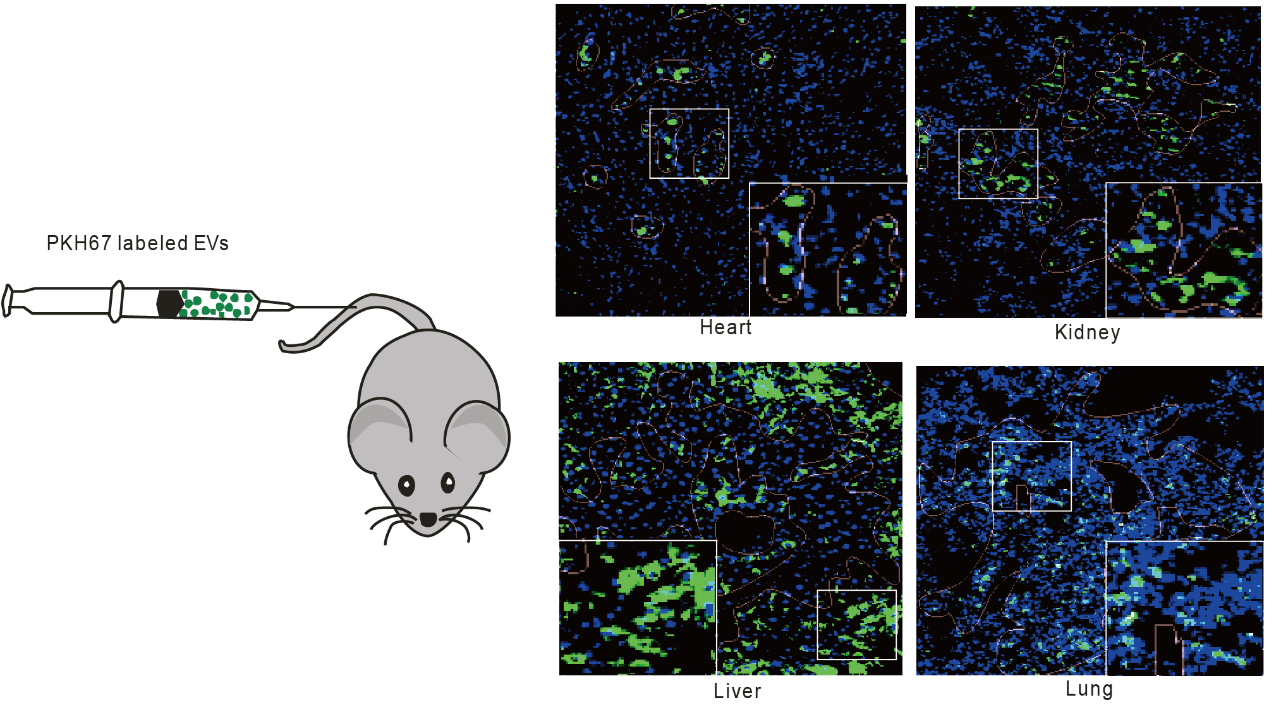


**Figure S2 In vivo uptake of PKH67 labeled EVs.** The PKH67-labeled Raw264.7-EVs (40μg) were transplanted into mouse liver by intravenous injection. After injection for 4 hours, the fresh tissues were embedded. Blocks were frozen and observed by confocal microscopy.
